# The relationship of indoxyl sulfate and p-cresyl sulfate with target cardiovascular proteins in hemodialysis patients

**DOI:** 10.1038/s41598-021-83383-x

**Published:** 2021-02-15

**Authors:** Ping-Hsun Wu, Yi-Ting Lin, Yi-Wen Chiu, Gabriel Baldanzi, Jiun-Chi Huang, Shih-Shin Liang, Su-Chu Lee, Szu-Chia Chen, Ya-Ling Hsu, Mei-Chuan Kuo, Shang-Jyh Hwang

**Affiliations:** 1 Graduate Institute of Clinical Medicine, College of Medicines, Kaohsiung Medical University, Kaohsiung, Taiwan; 2 Faculty of Medicine, College of Medicine, Kaohsiung Medical University, Kaohsiung, Taiwan; 3 Faculty of Renal Care, College of Medicine, Kaohsiung Medical University, Kaohsiung, Taiwan; 4 Department of Biotechnology, College of Life Science, Kaohsiung Medical University, Kaohsiung, Taiwan; 5 Graduate Institute of Medicine, College of Medicine, Kaohsiung Medical University, Kaohsiung, Taiwan; 6 Division of Nephrology, Department of Internal Medicine, Kaohsiung Medical University Hospital, 100 Shih-Chuan 1st Road, Kaohsiung, 807 Taiwan; 7 Department of Internal Medicine, Kaohsiung Municipal Siaogang Hospital, Kaohsiung Medical University, Kaohsiung, Taiwan; 8 Department of Family Medicine, Kaohsiung Medical University Hospital, Kaohsiung, Taiwan; 9 Department of Medical Sciences, Uppsala University, Uppsala, Sweden

**Keywords:** Haemodialysis, Molecular medicine, End-stage renal disease, Proteomic analysis

## Abstract

Protein-bound uremic toxins (Indoxyl sulfate [IS] and p-cresyl sulfate [PCS]) are both associated with cardiovascular (CV) and all-cause mortality in subjects with chronic kidney disease (CKD). Possible mechanisms have not been elucidated. In hemodialysis patients, we investigated the relationship between the free form of IS and PCS and 181 CV-related proteins. First, IS or PCS concentrations were checked, and high levels were associated with an increased risk of acute coronary syndrome (ACS) in 333 stable HD patients. CV proteins were further quantified by a proximity extension assay. We examined associations between the free form protein-bound uremic toxins and the quantified proteins with correction for multiple testing in the discovery process. In the second step, the independent association was evaluated by multivariable-adjusted models. We rank the CV proteins related to protein-bound uremic toxins by bootstrapped confidence intervals and ascending *p*-value. Six proteins (signaling lymphocytic activation molecule family member 5, complement component C1q receptor, C–C motif chemokine 15 [CCL15], bleomycin hydrolase, perlecan, and cluster of differentiation 166 antigen) were negatively associated with IS. Fibroblast growth factor 23 [FGF23] was the only CV protein positively associated with IS. Three proteins (complement component C1q receptor, CCL15, and interleukin-1 receptor-like 2) were negatively associated with PCS. Similar findings were obtained after adjusting for classical CV risk factors. However, only higher levels of FGF23 was related to increased risk of ACS. In conclusion, IS and PCS were associated with several CV-related proteins involved in endothelial barrier function, complement system, cell adhesion, phosphate homeostasis, and inflammation. Multiplex proteomics seems to be a promising way to discover novel pathophysiology of the uremic toxin.

## Introduction

Chronic kidney disease (CKD) is associated with high morbidity and mortality, mainly due to cardiovascular disease (CVD)^1^. Also, CVD is the leading cause of death in patients with end-stage renal disease (ESRD)^2^. Recently, non-traditional CVD risk factors, in particular uremic toxins, have been shown to contribute to CVD mortality and morbidity in hemodialysis (HD) patients^3^. Uremic toxins are biologically active and have a detrimental effect on the cardiovascular (CV) system, by affecting cells involved in myocardial and vessel functions, such as leucocytes, endothelial cells, smooth muscle cells, and platelets^4^. The accumulation of uremic toxins has been shown to cause vascular dysfunction in humans and experimental animals with CKD^5,6^.

Two protein-bound uremic toxins, indoxyl sulfate (IS) and p-cresyl sulfate (PCS), are the most well-investigated metabolites derived from the gut microbiota^7^. Pre-clinical studies have found that IS and PCS can activate the nuclear factor-kappaB (NF-kB) pathway, resulting in the production of pro-inflammatory cytokine and oxidative stress in renal tubular cells^8,9^. In CKD and HD patients, serum IS level has an inverse relationship with kidney function and a direct link with aortic calcification, pulse-wave velocity, first heart failure event, and all-cause and CV mortality^10^. Besides, the PCS level is an independent predictor of mortality^11,12^, CV events^6,13^, and vascular stiffening^14^ in patients with CKD.

The available evidence supports the adverse effects of uremic toxins in HD patients^7^; however, the mechanisms leading to CVD are not fully elucidated. Therefore, this study aimed to investigate the relationship between protein-bound uremic toxins and 184 CV-related proteins in ESRD patients undergoing hemodialysis. Understanding the impact of uremic toxins on the level of circulating CV proteins may provide insights into the pathophysiology of CVD in CKD patients.

## Methods

### Subjects, comorbidity, and biochemical measurements

We enrolled participants from dialysis units of Kaohsiung Medical University Hospital and Kaohsiung Municipal Hsiao-Kang Hospital between August 2016 and January 2017. Participants aged > 30 years and who received HD > 90 days were enrolled. All participants were under high-efficiency dialyzers therapy three times per week with an adequate dialysis target of Kt/V > 1.2. The HD settings were 250–300 ml/min of blood flow rate, 500 ml/min of dialysate flow, and 3.5–4 h of each session.

The baseline characteristics of HD patients were recorded from electronic healthcare record systems, including age, sex, dialysis vintage, arteriovenous access type (fistula or graft), the primary cause of kidney failure (hypertension, diabetes, glomerulonephritis, or others), comorbidities, medications, and biochemical data. Hypertension was defined as blood pressure over 140/90 mmHg or taking blood pressure-lowering drugs. Diabetes mellitus was defined as HbA1C of over 6.5% or taking glucose-lowering drugs. The biochemical data were obtained from routine blood samples within 30 days before protein-bound uremic toxins and CV proteomics measurement. We collected blood samples at the beginning of the week after overnight fasting from patients through the arteriovenous access immediately before the scheduled HD session and stored at − 80 °C.

### Ethical considerations

The Institutional Review Board approved the study of Kaohsiung Medical University (KMUHIRB-E(I)-20160095 and KMUHIRB-E(I)-20180139). The study was carried out following the Declaration of Helsinki. All subjects signed the informed consent form.

### Uremic toxins profiling

The free fraction of PCS and IS were analyzed by high-performance liquid chromatography with tandem mass spectrometry instrument (LC/MS–MS), as previously described^15^. The detailed process is described in Supplementary Methods (see Supplementary Materials).

### Proteomic profiling

Serum samples were assessed by the Proseek Multiplex 96 × 96 proximity extension assay using the CV II and III panel (Olink Bioscience, Uppsala, Sweden), as previously described^16^. Each panel simultaneously measures 92 proteins. The detailed methodology was described in Supplementary methods (see Supplementary Materials). During quality control, proteins with more than 15% of the values below the limit of detection (LOD) were removed. After excluding three proteins (natriuretic peptides B, melusin, and chitotriosidase-1), the final data included 181 proteins. The list of all proteins measured is in Supplementary Table 1.

### Acute coronary syndrome event definition

Patients were followed up for 2 years or until acute coronary syndrome (ACS) event. The ACS included ST-segment elevation-ACS (STE-ACS) and non-STE-ACS. ACS was diagnosed when any of the following conditions apply: (1) ECG findings met the criteria for a definitive diagnosis during an event; (2) cardiac ischemic chest pain was present, and enzyme levels met the criteria for a definitive diagnosis; (3) cardiac ischemic chest pain was present, enzyme levels met the criteria for suspected myocardial infarction, and ECG findings met the criteria for suspected myocardial infarction; (4) ECG findings, which met the criteria for a definitive diagnosis of myocardial infarction, were newly obtained on current ECG findings; and (5) elevated level of cardiac enzymes CK, CK-MB, and Troponin-I was measured during an event, and, of these enzymes, elevations greater than twofold the upper limit of normal were found in one or more.

### Statistical analysis

Continuous variables of demographic data are demonstrated as mean ± standard deviation (SD), and nominal variables are shown as percentages. In the discovery phase, the two protein-bound uremic toxins (IS and PCS) and 181 proteins were evaluated their association using linear regression models adjusting for age and sex. Associations significant at a false discovery rate (FDR) < 5% were further investigated in the next step. We calculate FDR by Benjamini and Hochberg's method^17^.

In the second analysis phase, we investigated the independent associations using multivariable-adjusted models. We selected the variables based on a causal diagram using the DAGitty software, version 2.2 (www.dagitty.net; Supplementary Fig. 1). The models included the covariates of age, sex, HD vintage, cause of ESRD, arteriovenous access type, diabetes mellitus, hypertension, dyslipidemia, antiplatelet/warfarin, antihypertensive drugs, diabetes treatment drugs, calcium, phosphate, high sensitivity C-reactive protein (hsCRP), and total Kt/V. Linearity associations between IS or PCS and CV proteins were further tested using restricted cubic splines with three knots. Also, we ranked the CV proteins related to IS or PCS by ascending *p*-value, with bootstrapped confidence intervals around the ranks. To determine the outcome association, Kaplan–Meier analysis was performed to calculate the cumulative incidence of ACS categorized according to the median value of IS, PCS, and protein biomarkers.

All statistical methods were performed using Stata (version 15, College Station, TX, USA). Results were presented as a beta coefficient (β) with a 95% confidence interval (CI). A two-tailed *p* < 0.05 was considered statistically significant in this second phase.

## Results

**Figure 1:**
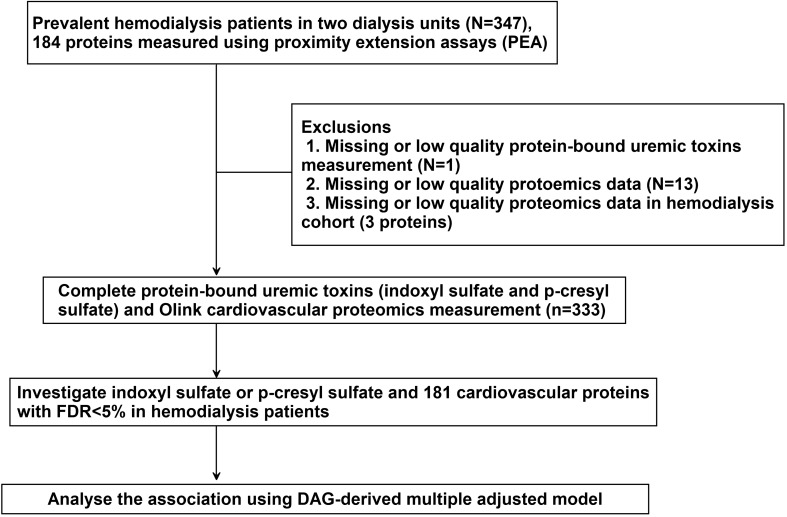
Study design.

Initially, 347 patients were enrolled. Subjects were excluded if the protein-bound uremic toxins measurements (n = 1) or the protein measurements (n = 13) were below the detection limit. Finally, the association between the two protein-bound uremic toxins (free forms of IS and PCS) and 181 proteins were analyzed in 333 patients (Fig. 1).

### Demographic and clinical characteristics

The patients’ characteristics were listed in Table 1. HD patients with CV comorbidities (coronary artery disease or cerebrovascular disease) were older, had a higher proportion of hypertension, diabetes mellitus, dyslipidemia, and more commonly used antiplatelets/warfarin, antihypertensive drugs, diabetes treatment drugs, and had higher hsCRP levels than patients without CV comorbidities.

**Table 1:** Baseline characteristics of hemodialysis participants with and without overt cardiovascular comorbidities (coronary artery disease or cerebrovascular disease).

	With cardiovascular comorbidities (n = 79)	Without cardiovascular comorbidities (n = 254)	*p* value
Age (years)	62.3 (11.1)	58.4 (11.6)	0.008
Male	45 (57.0)	132 (52.0)	0.437
Hemodialysis duration (years)	6.5 (5.14)	6.8 (5.81)	0.747
**Cause of ESRD**			
Hypertension	9 (11.4)	27 (10.6)	0.849
Diabetes mellitus	35 (44.3)	83 (32.7)	0.059
Glomerulonephritis	23 (29.1)	95 (37.4)	0.179
Others^a^	12 (15.2)	49 (19.3)	0.410
**Arteriovenous shunt**			
Arteriovenous fistula	65 (82.3)	226 (89.0)	0.117
Arteriovenous graft	14 (17.7)	28 (11.0)	0.117
**Comorbidities**			
Diabetes mellitus	42 (53.2)	103 (40.6)	0.048
Hypertension	70 (88.6)	188 (74.0)	0.007
Dyslipidemia	40 (50.6)	88 (34.6)	0.011
**Medications**			
Antiplatelets/Warfarin	53 (67.1)	42 (16.5)	< 0.001
Anti-hypertensive drugs	50 (64.1)	106 (41.9)	0.001
Diabetes treatment drugs	36 (46.2)	75 (29.6)	0.007
**Laboratory data**			
Ionized calcium (mg/dL)	4.7 (0.4)	4.6 (0.5)	0.651
Phosphate (mg/dL)	4.7 (1.1)	4.7 (1.1)	0.975
High sensitivity C-reactive protein (mg/L)	3.7 (6.6)	1.9 (3.1)	0.001
Total (Kt/V)	1.6 (0.2)	1.6 (0.3)	0.860
**Uremic toxins**			
Free form indoxyl sulfate (μg/mL)	1.9 (1.9)	1.9 (3.0)	0.984
Free form p-cresyl sulfate (μg/mL)	1.6 (2.0)	1.3 (1.7)	0.163

^a^Other causes of end-stage renal disease include polycystic kidney disease, tumor, systemic lupus erythematosus, gout, interstitial nephritis.

### Discovery phase

**Figure 2:**
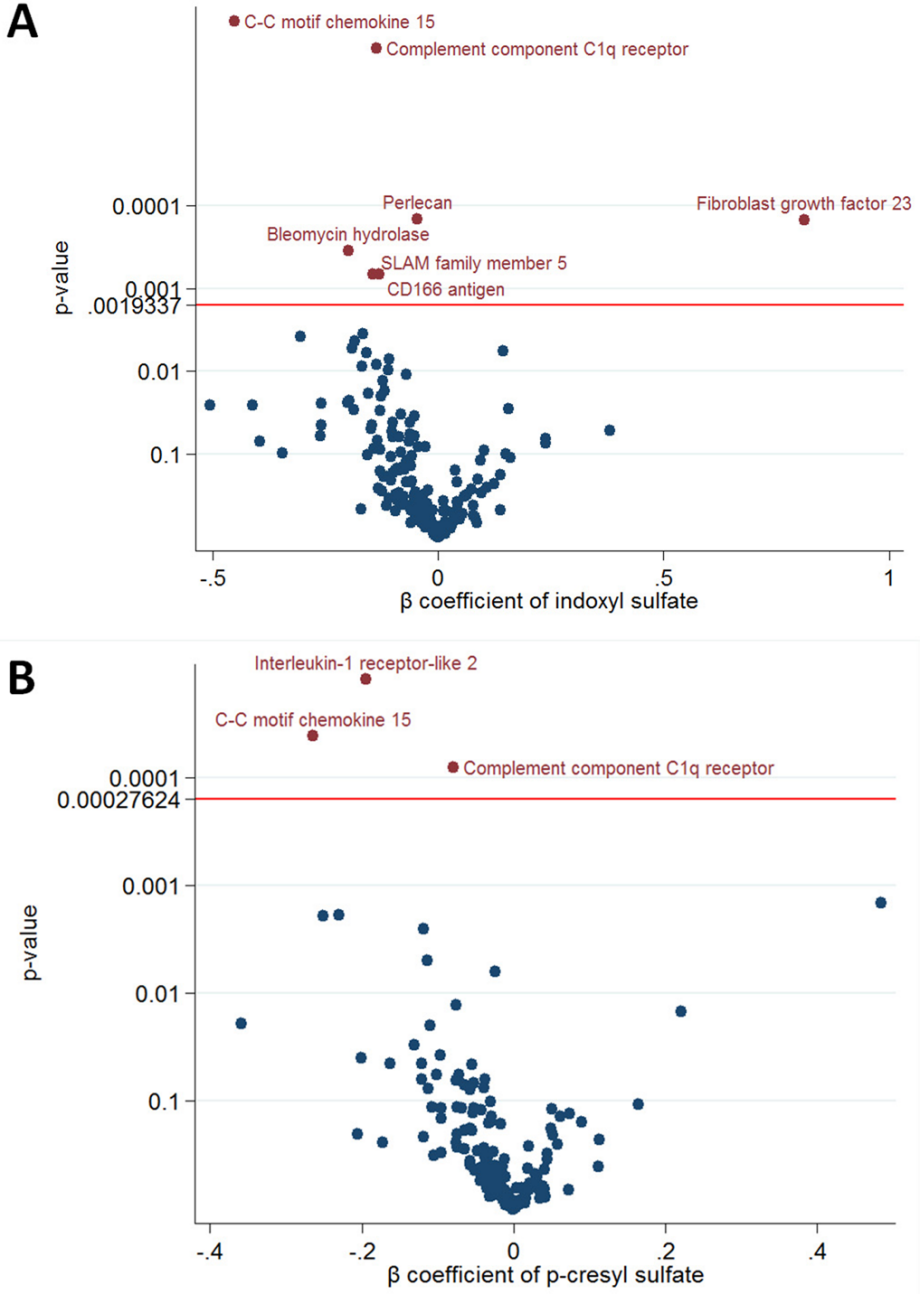
Volcano plot of the p-value and β coefficient for protein-bound uremic toxins and cardiovascular protein biomarkers association with multiple test control. (**A**) Indoxyl sulfate (**B**) p-cresyl sulfate.

The free form IS was significantly associated with seven proteins (C–C motif chemokine 15 [CCL15], complement component C1q receptor, perlecan, bleomycin hydrolase, cluster of differentiation 166 antigen [CD166], signaling lymphocytic activation molecule [SLAM] family member 5, and fibroblast growth factor 23 [FGF23]) (Fig. 2A). The free form PCS was significantly associated with three proteins (CCL15, complement component C1q receptor, and interleukin-1 receptor-like 2 [IL1RL2]) (Fig. 2B). The overall β coefficients and *p*-values for the associations between the uremic toxins (IS and PCS) and all proteins in HD patients are shown in Supplementary Figs. 2 and 3.

### Second analysis phase

Regarding the associations with the fully adjusted models, the free form IS was negatively associated with CCL15 (β − 0.53, 95% CI − 0.71 to − 0.35, *p* < 0.001), complement component C1q receptor (β − 0.14, 95% CI − 0.20 to − 0.09, *p* < 0.001), perlecan (β − 0.05, 95% CI − 0.07 to − 0.03, *p* < 0.001), bleomycin hydrolase (β − 0.22, 95% CI − 0.34 to − 0.11, *p* < 0.001), CD166 (β − 0.15, 95% CI − 0.23 to − 0.07, *p* < 0.001), and SLAM family member 5 (β − 0.13, 95% CI − 0.22 to − 0.05, *p* = 0.002), whereas free form IS was positively associated with FGF23 (β 0.63, 95% CI 0.23 to 1.04, *p* = 0.002) (Table 2). In the fully-adjusted model analysis, free form PCS was negatively associated with CCL15 (β − 0.29, 95% CI − 0.42 to − 0.16, *p* < 0.001), complement component C1q receptor (β − 0.07, 95% CI − 0.11 to − 0.03, *p* < 0.001), and IL1RL2 (β − 0.17, 95% CI − 0.26 to − 0.07, *p* < 0.001) (Table 3).

**Table 2:** Associations of free form indoxyl sulfate and selected cardiovascular protein biomarkers in the multivariate linear regression model.

	β coefficient (95% CI)	*p* value
C–C motif chemokine 15	− 0.53 (− 0.71 to − 0.35)	< 0.001
Complement component C1q receptor	− 0.14 (− 0.20 to − 0.09)	< 0.001
Perlecan	− 0.05 (− 0.07 to − 0.03)	< 0.001
Bleomycin hydrolase	− 0.22 (− 0.34 to − 0.11)	< 0.001
CD166 antigen	− 0.15 (− 0.23 to − 0.07)	< 0.001
SLAM family member 5	− 0.13 (− 0.22 to − 0.05)	0.002
Fibroblast growth factor 23	0.63 (0.23–1.04)	0.002

Associations of baseline free form indoxyl sulfate with selected protein biomarker NPX value using multivariate linear regression model adjusting for age, sex, hemodialysis duration, cause of end-stage renal disease, arteriovenous shunt type, comorbidities (diabetes mellitus, hypertension, and dyslipidemia), medications (antiplatelet/warfarin, anti-hypertensive drugs, diabetic treatment drugs), and laboratory data (calcium, phosphate, high sensitivity C-reactive protein, and Kt/V).

**Table 3:** Associations of free form p-cresyl sulfate and selected cardiovascular protein biomarkers in the multivariate linear regression model.

	β coefficient (95% CI)	*p* value
C–C motif chemokine 15	− 0.29 (− 0.42 to − 0.16)	< 0.001
Complement component C1q receptor	− 0.07 (− 0.11 to − 0.03)	< 0.001
Interleukin-1 receptor-like 2	− 0.16 (− 0.26 to − 0.07)	< 0.001

Associations of baseline free form p-cresyl sulfate with selected protein biomarker NPX value using multivariate linear regression model adjusting for age, sex, hemodialysis duration, cause of end-stage renal disease, arteriovenous shunt type, comorbidities (diabetes mellitus, hypertension, and dyslipidemia), medications (antiplatelet/warfarin, anti-hypertensive drugs, diabetic treatment drugs), and laboratory data (calcium, phosphate, high sensitivity C-reactive protein, and Kt/V).

Ranking of the associations (with 95% bootstrap-obtained CIs) of all proteins with the uremic toxins (IS or PCS) is graphically presented. Protein CCL15 and complement component C1q receptor were the top 2 hits related to IS (Supplementary Fig. 4). Regarding the associations with PCS, the top 3 hits were protein IL1RL2, CCL15, and complement component C1q (Supplementary Fig. 5). All the top hits had narrow confidence intervals of the rank.

**Figure 3:**
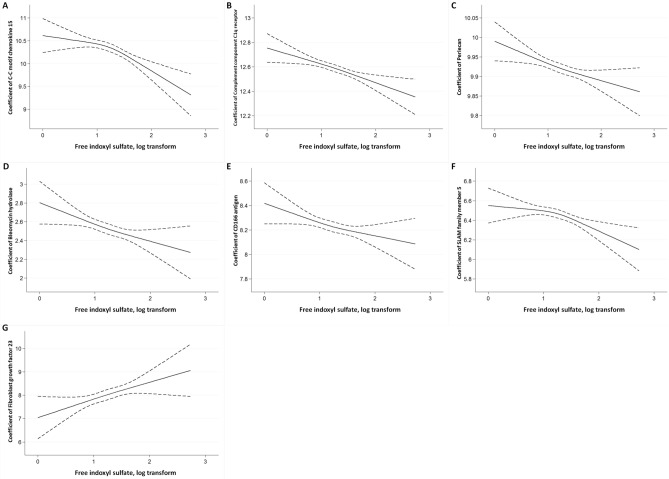
The cubic spline curve between the log-transformed indoxyl sulfate concentration with 3 knots and selected circulating cardiovascular protein biomarkers NPX units (**A**) C–C motif chemokine 15 (CCL15) (**B**) Complement component C1q receptor (CD93) (**C**) Perlecan (**D**) Bleomycin hydrolase (**E**) CD166 antigen (**F**) SLAM family member 5 (CD84) (**G**) Fibroblast growth factor 23 (FGF23).

**Figure 4:**
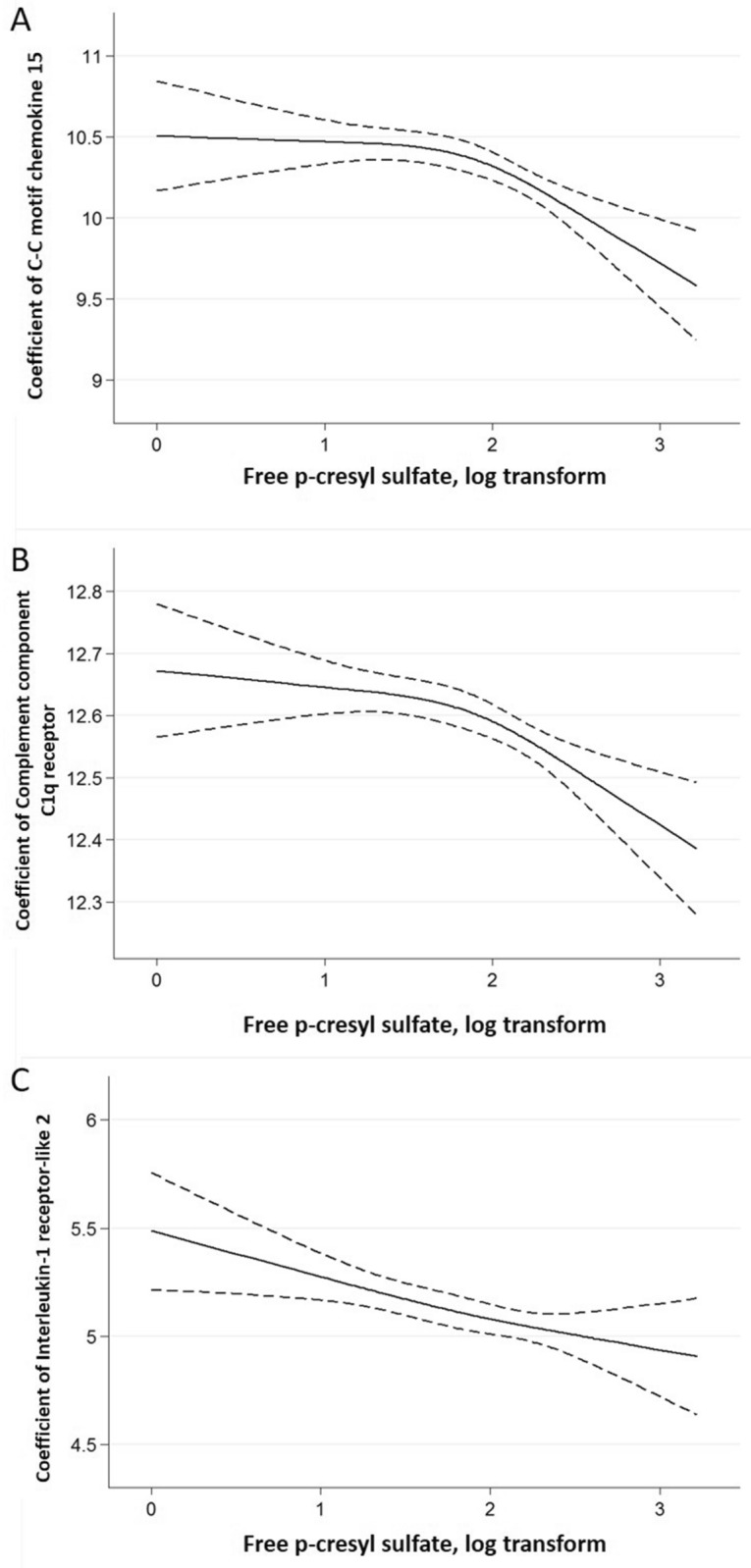
The cubic spline curve between the log-transformed p-cresyl sulfate concentration with 3 knots and selected circulating cardiovascular protein biomarkers NPX units (**A**) C–C motif chemokine 15 (CCL15) (**B**) Complement component C1q receptor (CD93) (**C**) Interleukin-1 receptor-like 2 (IL1RL2).

Cubic spline analysis demonstrated a linear association of IS with CCL15, complement component C1q receptor, perlecan, bleomycin hydrolase, CD166 antigen, SLAM family member 5, and FGF23 (Fig. 3), as well as a linear association of PCS with CCL15, complement component C1q receptor, and IL1RL2 (Fig. 4).

### Uremic toxins and CV proteins to determine the ACS outcome in 2-years

**Figure 5:**
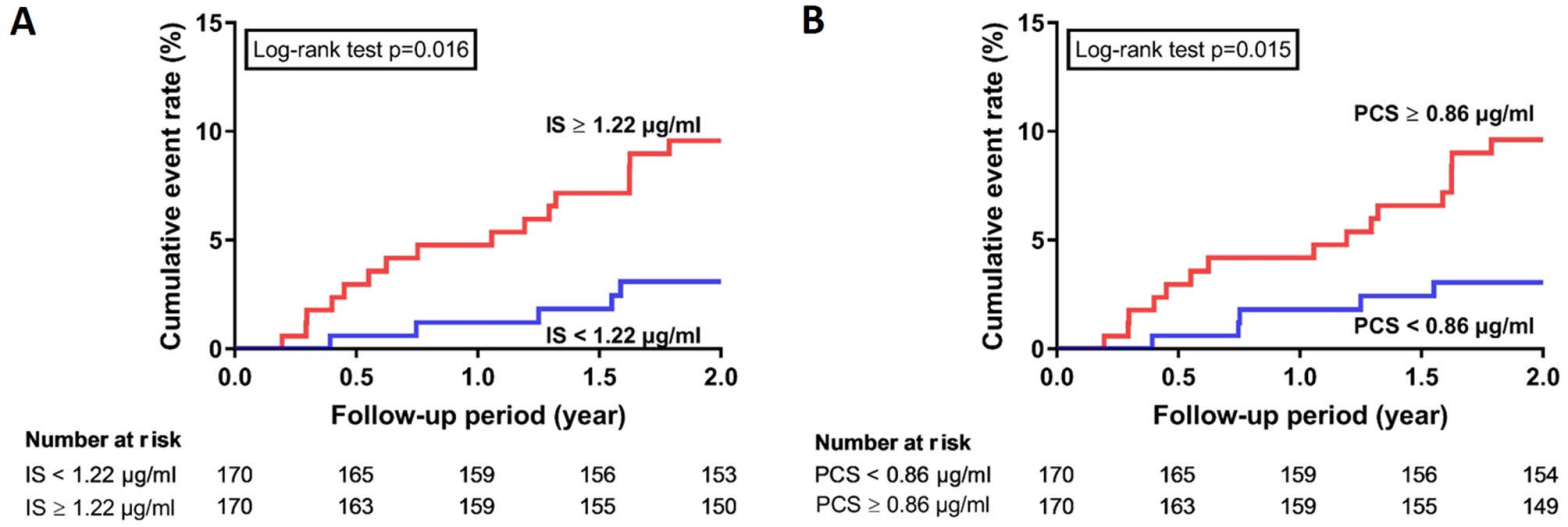
The Cumulative incidence of acute coronary syndrome in patients stratified by median level of protein-bound uremic toxins (**A**) Free form indoxyl sulfate (**B**) Free form p-cresyl sulfate.

The ACS outcome was assessed by the Kaplan–Meier method and log-rank test. The cumulative incidence of ACS was higher in the high IS group and PCS group than in the low IS group and low PCS group (Log-rank p = 0.016 in IS; Log-rank p = 0.015 in PCS) (Fig. 5). In contrast, there was no significant difference in selected CV protein biomarkers (CCL15, CD93, Perlecan, bleomycin hydrolase, CD166 antigen, CD84, and IL1RL2) between the two groups for the ACS outcomes (Supplementary Fig. 6). A higher level of FGF23 was found potential increased risks of ACS than a lower level of FGF23 (Log-rank p = 0.05) (Supplementary Fig. 6).

## Discussion

### Principal observations

In the present study, we investigated the associations of two protein-bound uremic toxins (IS and PCS) with 181 circulating protein biomarkers in a cohort of patients on HD treatment. After accounting for multiple testing controlled by FDR, free form IS levels were positively associated with FGF23 and negatively associated with CCL15, complement component C1q receptor, perlecan, bleomycin hydrolase, CD166 antigen, and SLAM family member 5. Furthermore, free form PCS levels were negatively associated with CCL15, complement component C1q receptor, and IL1RL2. This information can contribute to the knowledge regarding the underlying mechanism of non-traditional CVD risk factors in patients with kidney disease.

### The possible relationship of protein-bound uremic toxins with inflammation

Uremic toxins could promote cytokine expression and release in a pre-clinical study^18^. In the present study, the free forms of IS and PCS were negatively associated with CCL15, and free form PCS was negatively associated with IL1RL2.

CCL15, also known as leukotactin-1, is proinflammatory^19^ and involved in monocyte mediated atherosclerotic lesions^19–21^. Contrary to a previous report, this study found a negative association between the two protein-bound uremic toxins and CCL15^22^.

IL1RL2, also known as interleukin-36 (IL-36) receptor, is a member of the IL-1 receptor family. IL1RL2 can bind to IL-36, which is involved in several diseases, such as psoriasis^23^ or acute kidney injury^24^. Interestingly, genetically manipulated animal models lacking IL1RL2 are protected against the IL-36 triggered an inflammatory response in psoriasis^25,26^ and acute kidney injury models^24^. Thus, IL1RL2 could play a role in regulating inflammation. Hence, this negative association between PCS and IL1RL2 may highlight a possible pathway for the uremic toxins associated with CVD.

### Protein-bound uremic toxins, endothelial function, and the complement system

In this study, IS was negatively associated with perlecan, which along with β2 microglobulin, are co-localized in HD induced β-amyloidosis^27^. Perlecan is a large multi-domain proteoglycan that binds to extracellular matrix components and cell-surface molecules to maintain the endothelial barrier function^28^. It can stimulate endothelial growth and re-generation, inhibit smooth muscle cell proliferation, and help maintain vascular homeostasis^28,29^. Since perlecan is crucial for vascular repair regulation, the higher IS level associated with low perlecan may be another factor contributing to endothelial dysfunction.

We also demonstrated a negative association between free IS and bleomycin hydrolase in HD patients. Bleomycin hydrolase is an aminohydrolase involved in homocysteine metabolism, and defects in homocysteine metabolism can lead to hyperhomocysteinemia^30^. Pathological manifestations of hyperhomocysteinemia are associated with an increased risk of CV events in HD patients^31^. Furthermore, studies of genetic and nutritional hyperhomocysteinemia in animal models provide additional support for a causal role of homocysteine in atherothrombosis^32^. However, the regulation of IS on bleomycin hydrolase remains unknown.

CD166, also known as activated leukocyte cell adhesion molecule, is expressed on activated monocytes and endothelial cells, involved in cell migration processes^33,34^. The localization of CD166, specifically at cell–cell junctions on endothelial cells, supports its role in transendothelial migration^35^. CD166 can modulate endothelial function and is involved in the response of endothelial cells to inflammation^36^. Therefore, the shedding of CD166 may be impaired in high free IS conditions.

A negative association between uremic toxins (IS and PCS) and complement component C1q receptor was found in the current study. Complement component C1q receptor, known as CD93, plays a role in cell adhesion and phagocytosis in monocytes and macrophages^37^. It is over-expressed during inflammation, and the soluble form (soluble CD93) is increased in various inflammatory conditions. Soluble CD93 is associated with premature myocardial infarction, coronary artery disease, and other inflammatory conditions^38–40^, with reduced levels of the soluble CD93 related to metabolic dysregulation^41^. Thus, the release of soluble CD93 has been implicated in response to stressors, such as inflammatory, immune, and angiogenic mediators. However, the mechanism underlying the association between uremic toxins and soluble complement component C1q receptor remains unclear. Our finding proposes a link between protein-bound uremic toxins and complement component C1q receptor related to CVD.

IS could also induce thrombosis and atherosclerosis by enhancing platelet hyperactivity, elevating the response to collagen and thrombin, increasing platelet-derived microparticles and aggregation of platelet-monocytes^42^. The present study showed a positive association between IS and SLAM family member 5 (also known as CD84), which is expressed on the surface of platelets, and may stabilize platelet aggregation and thrombi via homophilic interactions^43^. In a previous clinical study, SLAM family member 5 was highly expressed in inflamed endothelial tissue of the coronary arteries of patients with Kawasaki disease^44^ and was also associated with early repeat coronary events in patients with acute coronary syndrome^45^. Thus, SLAM family member 5 could be involved with protein-bound uremic toxins related to platelet aggregation and thrombosis formation.

### Protein-bound uremic toxins, fibroblast growth factor 23/Klotho regulation, and cardiovascular damage

PCS and IS were positively associated with FGF23, but only the association with IS was significant (Supplementary Fig. 3). Several studies have confirmed the positive association between elevated serum FGF23 levels and mortality or CV events in HD patients^46,47^. Interestingly, the protein-bound uremic toxins and FGF23 association can link to their interference in the Klotho system. Klotho deficiency has been suggested as an upstream step of FGF23 excess, and IS-induced left ventricular hypertrophy (LVH) in Klotho-deficient mice was more severe^48^. By blocking oxidative stress, p38, and extracellular signal-regulated protein kinase 1/2 signaling pathways, exogenous Klotho could overwhelm IS-induced LVH in CKD mice^48^. In vitro and in vivo experiments showed that IS and PCS decreased Klotho expression in renal tubules by epigenetic silencing of the Klotho gene caused by oxidative stress^49^. The decrease in expression of Klotho can lead to a further compensatory increase in levels of FGF-23^50^, causing Klotho-independent adverse effects on off-target organs, such as pathological cardiac remodeling or increased hepatic production of inflammatory cytokines^51^.

### Potential biomarkers for ACS in HD patients

We determined an association between protein-bound uremic toxins (IS/PCS) and ACS risk, which was in line with previous studies^6,13,52–54^. Besides, we found a trend association between FGF23 and ACS. FGF23 was a well-established CV biomarker in HD patients^46,47^. However, other selected CV proteins (e.g., CCL15, CD93, Perlecan, bleomycin hydrolase, CD166 antigen, CD84, and IL1RL2) correlated to protein-bound uremic toxins were not found as biomarkers for ACS. Limited sample sizes and event numbers should be acknowledged in our study. More studies should be investigated to confirm our findings.

### Strengths and limitations

The advantages of this study include the comprehensive participant information and use of modern technology that allowed the rapid high-throughput, highly sensitive and specific analyses of limited plasma samples. The study limitations were that the cross-sectional study design precludes any causal inference, potentially introducing prevalence-incidence bias. Secondly, the selection of biomarkers used in this study was pragmatically restricted to CV-specific proteins, and it is possible that other proteins may be involved in the toxicity of IS or PCS. Further in vitro work is necessary to validate the translation of the mechanistic pathways suggested. Thirdly, only HD patients were recruited, so the findings may not be generalizable to other CKD or peritoneal dialysis populations. Fourthly, food intake was not evaluated to assess the intake of tryptophan, tyrosine, and fibers, which are the main sources of IS and PCS. Lastly, although PEA is a promising technique, improvements are warranted to assure clinically valid and reproducible measurements. Studying numerous proteins and protein-bound uremic toxins association may provide new insights on modulators of pathological CVD risk factors in HD patients, but the research remains exploratory. Thus, the proposed mechanisms through which biomarkers may be pathophysiologically related to IS and PCS are hypothesis-generating.

In conclusion, protein-bound uremic toxins (IS and PCS) were important factors for CVD in HD patients. These uremic toxins may contribute to inflammation, endothelial dysfunction, complement system, and FGF23 pathway, thereby providing insight into the mechanisms that lead to higher CVD mortality and death in HD patients. Strategies to reduce the accumulation of uremic toxins could reduce these CVD risk factors, thereby preventing CVD in HD patients.

## Supplementary Information

Supplementary Information.[Replace ESM file with the attached 'All Supplementary Tables and Figures (proofreading)_ESM

## Abbreviations

CKD: Chronic kidney disease
CVD: Cardiovascular disease
CV: Cardiovascular
IS: Indoxyl sulfate
PCS: P-cresyl sulfate
HD: Hemodialysis
LOD: Limit of detection
FDR: False discovery rate
hsCRP: High sensitivity C-reactive protein
SD: Standard deviation
CI: Confidence interval
CCL15: C–C motif chemokine 15
CD166: Cluster of differentiation 166 antigen
SLAM: Signaling lymphocytic activation molecule
FGF23: Fibroblast growth factor 23
IL1RL2: Complement component C1q receptor, and interleukin-1 receptor-like 2

## References

[1] Gansevoort RT (2013). Chronic kidney disease and cardiovascular risk: Epidemiology, mechanisms, and prevention. Lancet.

[2] Gargiulo R, Suhail F, Lerma EV (2015). Cardiovascular disease and chronic kidney disease. Dis. Mon..

[3] Cozzolino M (2018). Cardiovascular disease in dialysis patients. Nephrol. Dial. Transpl..

[4] Moradi H, Sica DA, Kalantar-Zadeh K (2013). Cardiovascular burden associated with uremic toxins in patients with chronic kidney disease. Am. J. Nephrol..

[5] Meijers BK (2009). The uremic retention solute p-cresyl sulfate and markers of endothelial damage. Am. J. Kidney Dis..

[6] Meijers BK (2010). p-Cresol and cardiovascular risk in mild-to-moderate kidney disease. Clin. J. Am. Soc. Nephrol..

[7] Mair RD, Sirich TL, Meyer TW (2018). Uremic toxin clearance and cardiovascular toxicities. Toxins (Basel).

[8] Shimizu H (2011). NF-kappaB plays an important role in indoxyl sulfate-induced cellular senescence, fibrotic gene expression, and inhibition of proliferation in proximal tubular cells. Am. J. Physiol. Cell Physiol..

[9] Watanabe H (2013). p-Cresyl sulfate causes renal tubular cell damage by inducing oxidative stress by activation of NADPH oxidase. Kidney Int..

[10] Barreto FC (2009). Serum indoxyl sulfate is associated with vascular disease and mortality in chronic kidney disease patients. Clin. J. Am. Soc. Nephrol..

[11] Bammens B, Evenepoel P, Keuleers H, Verbeke K, Vanrenterghem Y (2006). Free serum concentrations of the protein-bound retention solute p-cresol predict mortality in hemodialysis patients. Kidney Int..

[12] Liabeuf S (2010). Free p-cresylsulphate is a predictor of mortality in patients at different stages of chronic kidney disease. Nephrol. Dial Transpl..

[13] Lin CJ (2010). Serum protein-bound uraemic toxins and clinical outcomes in haemodialysis patients. Nephrol. Dial Transpl..

[14] Rossi M (2014). Protein-bound uremic toxins, inflammation and oxidative stress: a cross-sectional study in stage 3–4 chronic kidney disease. Arch. Med. Res..

[15] Lin YT (2019). Protein-bound uremic toxins are associated with cognitive function among patients undergoing maintenance hemodialysis. Sci. Rep..

[16] Wu PH (2019). Exploring the benefit of 2-methylbutyric acid in patients undergoing hemodialysis using a cardiovascular proteomics approach. Nutrients.

[17] Yoav HYB (1995). Controlling the false discovery rate: A practical and powerful approach to multiple testing. J. R. Stat. Soc. Series B Methodol..

[18] Nakano T (2019). Uremic toxin indoxyl sulfate promotes proinflammatory macrophage activation via the interplay of OATP2B1 and Dll4-Notch signaling. Circulation.

[19] Lee WH (2002). A novel chemokine, Leukotactin-1, induces chemotaxis, pro-atherogenic cytokines, and tissue factor expression in atherosclerosis. Atherosclerosis.

[20] Kwon SH (2008). Chemokine Lkn-1/CCL15 enhances matrix metalloproteinase-9 release from human macrophages and macrophage-derived foam cells. Nutr. Res. Pract..

[21] Yu R (2004). Involvement of leukotactin-1, a novel CC chemokine, in human atherosclerosis. Atherosclerosis.

[22] Richter R (2006). Increase of expression and activation of chemokine CCL15 in chronic renal failure. Biochem. Biophys. Res. Commun..

[23] Dietrich D, Gabay C (2014). Inflammation: IL-36 has proinflammatory effects in skin but not in joints. Nat. Rev. Rheumatol..

[24] Nishikawa H (2018). Knockout of the interleukin-36 receptor protects against renal ischemia-reperfusion injury by reduction of proinflammatory cytokines. Kidney Int..

[25] Barton JL, Herbst R, Bosisio D, Higgins L, Nicklin MJ (2000). A tissue specific IL-1 receptor antagonist homolog from the IL-1 cluster lacks IL-1, IL-1ra, IL-18 and IL-18 antagonist activities. Eur. J. Immunol..

[26] Tortola L (2012). Psoriasiform dermatitis is driven by IL-36-mediated DC-keratinocyte crosstalk. J. Clin. Invest..

[27] Ohashi K (2001). Pathogenesis of beta2-microglobulin amyloidosis. Pathol. Int..

[28] Iozzo RV (1998). Matrix proteoglycans: From molecular design to cellular function. Annu. Rev. Biochem..

[29] Forsten KE, Courant NA, Nugent MA (1997). Endothelial proteoglycans inhibit bFGF binding and mitogenesis. J. Cell. Physiol..

[30] Suszynska-Zajczyk J (2014). Bleomycin hydrolase and hyperhomocysteinemia modulate the expression of mouse proteins involved in liver homeostasis. Amino Acids.

[31] Heinz J, Kropf S, Luley C, Dierkes J (2009). Homocysteine as a risk factor for cardiovascular disease in patients treated by dialysis: A meta-analysis. Am. J. Kidney. Dis..

[32] Borowczyk K, Tisonczyk J, Jakubowski H (2012). Metabolism and neurotoxicity of homocysteine thiolactone in mice: Protective role of bleomycin hydrolase. Amino Acids.

[33] Ohneda O (2001). ALCAM (CD166): Its role in hematopoietic and endothelial development. Blood.

[34] Sun Y (2015). Expression and role of CD166 in the chronic kidney disease. Iran J. Pediatr..

[35] Masedunskas A (2006). Activated leukocyte cell adhesion molecule is a component of the endothelial junction involved in transendothelial monocyte migration. FEBS Lett..

[36] Ikeda K, Quertermous T (2004). Molecular isolation and characterization of a soluble isoform of activated leukocyte cell adhesion molecule that modulates endothelial cell function. J. Biol. Chem..

[37] Greenlee-Wacker MC, Galvan MD, Bohlson SS (2012). CD93: Recent advances and implications in disease. Curr. Drug Targets.

[38] Greenlee MC, Sullivan SA, Bohlson SS (2009). Detection and characterization of soluble CD93 released during inflammation. Inflamm. Res..

[39] Jeon JW (2010). Soluble CD93 induces differentiation of monocytes and enhances TLR responses. J. Immunol..

[40] Malarstig A (2011). Plasma CD93 concentration is a potential novel biomarker for coronary artery disease. J. Intern. Med..

[41] Strawbridge RJ (2016). Soluble CD93 is involved in metabolic dysregulation but does not influence carotid intima-media thickness. Diabetes.

[42] Yang K (2017). Indoxyl sulfate induces platelet hyperactivity and contributes to chronic kidney disease-associated thrombosis in mice. Blood.

[43] Nanda N (2005). Platelet aggregation induces platelet aggregate stability via SLAM family receptor signaling. Blood.

[44] Reindel R (2014). CD84 is markedly up-regulated in Kawasaki disease arteriopathy. Clin. Exp. Immunol..

[45] Vroegindewey MM (2019). The temporal pattern of immune and inflammatory proteins prior to a recurrent coronary event in post-acute coronary syndrome patients. Biomarkers.

[46] Isakova T (2011). Fibroblast growth factor 23 and risks of mortality and end-stage renal disease in patients with chronic kidney disease. JAMA.

[47] Gao S, Xu J, Zhang S, Jin J (2019). Meta-analysis of the association between fibroblast growth factor 23 and mortality and cardiovascular events in hemodialysis patients. Blood Purif..

[48] Yang K (2015). Klotho protects against indoxyl sulphate-induced myocardial hypertrophy. J. Am. Soc. Nephrol..

[49] Sun CY, Chang SC, Wu MS (2012). Suppression of Klotho expression by protein-bound uremic toxins is associated with increased DNA methyltransferase expression and DNA hypermethylation. Kidney Int..

[50] Kuro-o M (2012). Klotho in health and disease. Curr. Opin. Nephrol. Hypertens..

[51] Richter B, Faul C (2018). FGF23 actions on target tissues-with and without Klotho. Front. Endocrinol. (Lausanne).

[52] Lin CJ (2012). Indoxyl sulfate predicts cardiovascular disease and renal function deterioration in advanced chronic kidney disease. Arch. Med. Res..

[53] Meijers BK (2008). Free p-cresol is associated with cardiovascular disease in hemodialysis patients. Kidney Int..

[54] Poesen R (2014). Cardiovascular disease relates to intestinal uptake of p-cresol in patients with chronic kidney disease. BMC Nephrol..

