# Supplementary material for: The relationship of indoxyl sulfate and p-cresyl sulfate with target cardiovascular proteins in hemodialysis patients
Source: Sci Rep. 2021 Feb 15;11:3786. doi: 10.1038/s41598-021-83383-x (PMC7884394; doi:10.1038/s41598-021-83383-x)

**The relationship of indoxyl sulfate and p-cresyl sulfate with target cardiovascular proteins in hemodialysis patients**

Ping-Hsun Wu^1,2,6,9^, Yi-Ting Lin^1,2,8,9^, Yi-Wen Chiu^2,3,6^, Gabriel Baldanzi^9^, Jiun-Chi Huang^1,2,6,7^, Shih-Shin Liang^4^, Su-Chu Lee^6^, Szu-Chia Chen^1,2,6,7^, Ya-Ling Hsu^5^, Mei-Chuan Kuo^2,3,6^, Shang-Jyh Hwang^2,3,6^

^1^Graduate Institute of Clinical Medicine, College of Medicines, Kaohsiung Medical University, Kaohsiung, Taiwan

^2^Faculty of Medicine, College of Medicine, Kaohsiung Medical University, Kaohsiung, Taiwan

^3^Faculty of Renal Care, College of Medicine, Kaohsiung Medical University, Kaohsiung, Taiwan

^4^Department of Biotechnology, College of Life Science, Kaohsiung Medical University, Kaohsiung, Taiwan

^5^Graduate Institute of Medicine, College of Medicine, Kaohsiung Medical University, Kaohsiung, Taiwan

^6^Division of Nephrology, Department of Internal Medicine, Kaohsiung Medical University Hospital, Kaohsiung, Taiwan

^7^Department of Internal Medicine, Kaohsiung Municipal Siaogang Hospital, Kaohsiung Medical University, Kaohsiung, Taiwan

^8^Department of Family Medicine, Kaohsiung Medical University Hospital, Kaohsiung, Taiwan

^9^Department of Medical Sciences, Uppsala University, Uppsala, Sweden

**Corresponding Author**

Mei-Chuan Kuo, MD

Division of Nephrology, Department of Internal Medicine, Kaohsiung Medical University Hospital, Kaohsiung, Taiwan

100 Shih-Chuan 1st Road Kaohsiung 807, Taiwan

Telephone number: 886-7-3121101 ext 7351

Fax number: 886-7-3228721

E-mail address: [mechku@kmu.edu.tw](mailto:mechku@kmu.edu.tw)

**Supplemental Methods.**

**Protein-bound uremic toxins measurement**

The free fraction of IS and PCS were analyzed using the tandem MS system that equipped with an Acella 1250 Ultra-high performance liquid chromatography (UHPLC) analytical system (Thermo Fisher Scientific Inc., Waltham, MA, USA) and micro electrospray ionization (ESI) ion source, as previously described.^1^ In brief, each serum sample (300 μL) was filled into a centrifugal filter device (Amico Ultra 3K, MerckMillipore), and centrifuged for 30 min at 13,300 g at 4℃. The supernatant was then evaporated with a spin vacuum instrument. The lyophilized samples were re-dissolved with 100 µL 30% acetonitrile (MeCN) with 0.1% formic acid and were added 10 µL IS-d4 (internal standard; Sigma-Aldrich, 1000 ng/mL) for each sample for further MS analysis. The negative ion mode was set with a voltage of 2.5 kV as the detection mode, with vaporizing and capillary temperatures of 300°C and 350°C, respectively. The quantification was evaluated by scan mode multiple reaction monitoring (MRM) transitions 212>80 and 212>132 for IS and 187>80 and 187>107 for PCS. The MS spectra and control of the mass spectrometer was via Xcalibur software (version 2.2, Thermo-Finnigan Inc., San Jose, CA, USA).

**Cardiovascular proteomics measurement**

The Proseek Multiplex 96 x 96 proximity extension assay simultaneously measured 184 proteins (Supplementary Table 1), as previously described.^2^ In brief, two protein-specific antibodies that attached to oligonucleotide strands was used. Each sample contained two incubations, one extension, and one detection control used to determine the lower detection limit and normalize the measurements. When both antibodies are bound to the target protein, the oligonucleotides are brought together and amplified in a quantitative polymerase chain reaction (qPCR). The relative concentration of the target protein was correlated to the qPCR values. Normalized protein expression (NPX) values were generated from qPCR quantification cycle values by log_2_-transformation. The NPX values were corrected for technical variation by an interplate control and determined limited of detection via a negative control. Mean intra-assay and inter-assay coefficients of variation were 4% and 10%, respectively. Quality control was performed to remove of proteins with >15% samples below the limited of detection and subjects with a high proportion of missing protein values.

Reference List

1 Lin, Y. T. *et al.* Protein-bound uremic toxins are associated with cognitive function among patients undergoing maintenance hemodialysis. *Sci Rep* **9**, 20388, doi:10.1038/s41598-019-57004-7 (2019).

2 Wu, P. H. *et al.* Exploring the Benefit of 2-Methylbutyric Acid in Patients Undergoing Hemodialysis Using a Cardiovascular Proteomics Approach. *Nutrients* **11**, doi:10.3390/nu11123033 (2019).

**Supplementary Table 1.** List of 184 proteins measured by proximity extension assay. The 3 proteins that failed quality control metrics were excluded from the analysis as indicated in the fourth column

| Abbreviation | Protein Analyte | UniProt ID | Excluded from Analysis |
| --- | --- | --- | --- |
| ACE2 | Angiotensin-converting enzyme 2 | Q9BYF1 |  |
| ADAM-TS13 | A disintegrin and metalloproteinase with thrombospondin motifs 13 | Q76LX8 |  |
| ADM | ADM | P35318 |  |
| AGRP | Agouti-related protein | O00253 |  |
| AMBP | Protein AMBP | P02760 |  |
| ANG-1 | Angiopoietin-1 | Q15389 |  |
| BMP-6 | Bone morphogenetic protein 6 | P22004 |  |
| BNP | Natriuretic peptides B | P16860 | Y |
| BOC | Brother of CDO | Q9BWV1 |  |
| CA5A | Carbonic anhydrase 5A, mitochondrial | P35218 |  |
| CCL17 | C-C motif chemokine 17 | Q92583 |  |
| CCL3 | C-C motif chemokine 3 | P10147 |  |
| CD4 | T-cell surface glycoprotein CD4 | P01730 |  |
| CD40-L | CD40 ligand | P29965 |  |
| CD84 | SLAM family member 5 | Q9UIB8 |  |
| CEACAM8 | Carcinoembryonic antigenrelated cell adhesion molecule 8 | P31997 |  |
| CTRC | Chymotrypsin C | Q99895 |  |
| CTSL1 | Cathepsin L1 | P07711 |  |
| CXCL1 | C-X-C motif chemokine 1 | P09341 |  |
| DCN | Decorin | P07585 |  |
| DECR1 | 2,4-dienoyl-CoA reductase, mitochondrial | Q16698 |  |
| Dkk-1 | Dickkopf-related protein 1 | O94907 |  |
| FABP2 | Fatty acid-binding protein, intestinal | P12104 |  |
| FGF-21 | Fibroblast growth factor 21 | Q9NSA1 |  |
| FGF-23 | Fibroblast growth factor 23 | Q9GZV9 |  |
| FS | Follistatin | P19883 |  |
| Gal-9 | Galectin-9 | O00182 |  |
| GDF-2 | Growth/differentiation factor 2 | Q9UK05 |  |
| GH | Growth hormone | P01241 |  |
| GIF | Gastric intrinsic factor | P27352 |  |
| GLO1 | Lactoylglutathione lyase | Q04760 |  |
| GT | Gastrotropin | P51161 |  |
| HAOX1 | Hydroxy acid oxidase 1 | Q9UJM8 |  |
| HB-EGF | Proheparin-binding EGF-like growth factor | Q99075 |  |
| HO-1 | Heme oxygenase 1 | P09601 |  |
| hOSCAR | Osteoclast-associated immunoglobulin-like receptor | Q8IYS5 |  |
| HSP 27 | Heat shock 27 kDa protein | P04792 |  |
| IDUA | Alpha-L-iduronidase | P35475 |  |
| IgG Fc receptor II-b | Low-affinity immunoglobulin gamma Fc region receptor II-b | P31994 |  |
| IL16 | Pro-interleukin-16 | Q14005 |  |
| IL-17D | Interleukin-17D | Q8TAD2 |  |
| IL18 | Interleukin-18 | Q14116 |  |
| IL-1ra | Interleukin-1 receptor antagonist protein | P18510 |  |
| IL1RL2 | Interleukin-1 receptor-like 2 | Q9HB29 |  |
| IL-27 | Interleukin-27 | Q8NEV9,Q14213 |  |
| IL-4RA | Interleukin-4 receptor subunit alpha | P24394 |  |
| IL6 | Interleukin-6 | P05231 |  |
| ITGB1BP2 | Melusin | Q9UKP3 | Y |
| KIM1 | Kidney Injury Molecule | Q96D42 |  |
| LEP | Leptin | P41159 |  |
| LOX-1 | Lectin-like oxidized LDL receptor 1 | P78380 |  |
| LPL | Lipoprotein lipase | P06858 |  |
| MARCO | Macrophage receptor MARCO | Q9UEW3 |  |
| MERTK | Tyrosine-protein kinase Mer | Q12866 |  |
| MMP12 | Matrix metalloproteinase-12 | P39900 |  |
| MMP7 | Matrix metalloproteinase-7 | P09237 |  |
| NEMO | NF-kappa-B essential modulator | Q9Y6K9 |  |
| PAPPA | Pappalysin-1 | Q13219 |  |
| PAR-1 | Proteinase-activated receptor 1 | P25116 |  |
| PARP-1 | Poly [ADP-ribose] polymerase 1 | P09874 |  |
| PDGF subunit B | Platelet-derived growth factor subunit B | P01127 |  |
| PD-L2 | Programmed cell death 1 ligand 2 | Q9BQ51 |  |
| PGF | Placenta growth factor | P49763 |  |
| PIgR | Polymeric immunoglobulin receptor | P01833 |  |
| PRELP | Prolargin | P51888 |  |
| PRSS27 | Serine protease 27 | Q9BQR3 |  |
| PRSS8 | Prostasin | Q16651 |  |
| PSGL-1 | P-selectin glycoprotein ligand 1 | Q14242 |  |
| PTX3 | Pentraxin-related protein PTX3 | P26022 |  |
| RAGE | Receptor for advanced glycosylation end products | Q15109 |  |
| REN | Renin | P00797 |  |
| SCF | Stem cell factor | P21583 |  |
| SERPINA12 | Serpin A12 | Q8IW75 |  |
| SLAMF7 | SLAM family member 7 | Q9NQ25 |  |
| SOD2 | Superoxide dismutase [Mn], mitochondrial | P04179 |  |
| SORT1 | Sortilin | Q99523 |  |
| SPON2 | Spondin-2 | Q9BUD6 |  |
| SRC | Proto-oncogene tyrosine-protein kinase Src | P12931 |  |
| STK4 | Serine/threonine-protein kinase 4 | Q13043 |  |
| TF | Tissue factor | P13726 |  |
| TGM2 | Protein-glutamine gamma-glutamyltransferase 2 | P21980 |  |
| THBS2 | Thrombospondin-2 | P35442 |  |
| THPO | Thrombopoietin | P40225 |  |
| TIE2 | Angiopoietin-1 receptor | Q02763 |  |
| TM | Thrombomodulin TM | P07204 |  |
| TNFRSF10A | Tumor necrosis factor receptor superfamily member 10A | O00220 |  |
| TNFRSF11A | Tumor necrosis factor receptor superfamily member 11A | Q9Y6Q6 |  |
| TNFRSF13B | Tumor necrosis factor receptor superfamily member 13B | O14836 |  |
| TRAIL-R2 | TNF-related apoptosis-inducing ligand receptor 2 | O14763 |  |
| VEGFD | Vascular endothelial growth factor D | O43915 |  |
| VSIG2 | V-set and immunoglobulin domain-containing protein 2 | Q96IQ7 |  |
| XCL1 | Lymphotactin | P47992 |  |
| ALCAM | Aminopeptidase N | Q13740 |  |
| AP-N | Azurocidin | P15144 |  |
| AXL | CD166 antigen | P30530 |  |
| AZU1 | Tyrosine-protein kinase receptor UFO | P20160 |  |
| BLM hydrolase | Bleomycin hydrolase | Q13867 |  |
| CASP-3 | Caspase-3 | P42574 |  |
| CCL15 | C-C motif chemokine 15 | Q16663 |  |
| CCL16 | C-C motif chemokine 16 | O15467 |  |
| CCL24 | C-C motif chemokine 24 | O00175 |  |
| CD163 | Scavenger receptor cysteine-rich type 1 protein M130 | Q86VB7 |  |
| CD93 | Complement component C1q receptor | Q9NPY3 |  |
| CDH5 | Cadherin-5 | P33151 |  |
| CHI3L1 | Chitinase-3-like protein 1 | P36222 |  |
| CHIT1 | Chitotriosidase-1 | Q13231 | Y |
| CNTN1 | Contactin-1 | Q12860 |  |
| COL1A1 | Collagen alpha-1(I) chain | P02452 |  |
| CPA1 | Carboxypeptidase A1 | P15085 |  |
| CPB1 | Carboxypeptidase B | P15086 |  |
| CSTB | Cystatin-B | P04080 |  |
| CTSD | Cathepsin D | P07339 |  |
| CTSZ | Cathepsin Z | Q9UBR2 |  |
| CXCL16 | C-X-C motif chemokine 16 | Q9H2A7 |  |
| DLK-1 | Protein delta homolog 1 | P80370 |  |
| EGFR | Epidermal growth factor receptor | P00533 |  |
| Ep-CAM | Epithelial cell adhesion molecule | P16422 |  |
| EPHB4 | Ephrin type-B receptor 4 | P54760 |  |
| FABP4 | Fatty acid-binding protein, adipocyte | P15090 |  |
| FAS | Tumor necrosis factor receptor superfamily member 6 | P25445 |  |
| Gal-3 | Galectin-3 | P17931 |  |
| Gal-4 | Galectin-4 | P56470 |  |
| GDF-15 | Growth/differentiation factor 15 | Q99988 |  |
| GP6 | Platelet glycoprotein VI | Q9HCN6 |  |
| GRN | Granulins | P28799 |  |
| ICAM-2 | Intercellular adhesion molecule 2 | P13598 |  |
| IGFBP-1 | Insulin-like growth factor-binding protein 1 | P08833 |  |
| IGFBP-2 | Insulin-like growth factor-binding protein 2 | P18065 |  |
| IGFBP-7 | Insulin-like growth factor-binding protein 7 | Q16270 |  |
| IL-17RA | Interleukin-17 receptor A | Q96F46 |  |
| IL-18BP | Interleukin-18-binding protein | O95998 |  |
| IL-1RT1 | Interleukin-1 receptor type 1 | P14778 |  |
| IL-1RT2 | Interleukin-1 receptor type 2 | P27930 |  |
| IL2-RA | Interleukin-2 receptor subunit alpha | P01589 |  |
| IL-6RA | Interleukin-6 receptor subunit alpha | P08887 |  |
| ITGB2 | Integrin beta-2 | P05107 |  |
| JAM-A | Junctional adhesion molecule A | Q9Y624 |  |
| KLK6 | Kallikrein-6 | Q92876 |  |
| LDL receptor | Low-density lipoprotein receptor | P01130 |  |
| LTBR | Lymphotoxin-beta receptor | P36941 |  |
| MB | Myoglobin | P02144 |  |
| MCP-1 | Monocyte chemotactic protein 1 | P13500 |  |
| MEPE | Matrix extracellular phosphoglycoprotein | Q9NQ76 |  |
| MMP-2 | Matrix metalloproteinase-2 | P08253 |  |
| MMP-3 | Matrix metalloproteinase-3 | P08254 |  |
| MMP-9 | Matrix metalloproteinase-9 | P14780 |  |
| MPO | Myeloperoxidase | P05164 |  |
| Notch 3 | Neurogenic locus notch homolog protein 3 | Q9UM47 |  |
| NT-proBNP | N-terminal prohormone brain natriuretic peptide | NA |  |
| OPG | Osteoprotegerin | O00300 |  |
| OPN | Osteopontin | P10451 |  |
| PAI | Plasminogen activator inhibitor 1 | P05121 |  |
| PCSK9 | Proprotein convertase subtilisin/kexin type 9 | Q8NBP7 |  |
| PDGF subunit A | Platelet-derived growth factor subunit A | P04085 |  |
| PECAM-1 | Platelet endothelial cell adhesion molecule | P16284 |  |
| PGLYRP1 | Peptidoglycan recognition protein 1 | O75594 |  |
| PI3 | Elafin | P19957 |  |
| PLC | Perlecan | P98160 |  |
| PON3 | Paraoxonase | Q15166 |  |
| PRTN3 | Myeloblastin | P24158 |  |
| PSP-D | Pulmonary surfactant-associated protein D | P35247 |  |
| RARRES2 | Retinoic acid receptor responder protein 2 | Q99969 |  |
| RETN | Resistin | Q9HD89 |  |
| SCGB3A2 | Secretoglobin family 3A member 2 | Q96PL1 |  |
| SELE | E-selectin | P16581 |  |
| SELP | P-selectin | P16109 |  |
| SHPS-1 | Tyrosine-protein phosphatase non-receptor type substrate 1 | P78324 |  |
| SPON1 | Spondin-1 | Q9HCB6 |  |
| ST2 | ST2 protein | Q01638 |  |
| TFF3 | Trefoil factor 3 | Q07654 |  |
| TFPI | Tissue factor pathway inhibitor | P10646 |  |
| TIMP4 | Metalloproteinase inhibitor 4 | Q99727 |  |
| TLT-2 | Trem-like transcript 2 protein | Q5T2D2 |  |
| TNF-R1 | Tumor necrosis factor receptor 1 | P19438 |  |
| TNF-R2 | Tumor necrosis factor receptor 2 | P20333 |  |
| TNFRSF10C | Tumor necrosis factor receptor superfamily member 10C | O14798 |  |
| TNFRSF14 | Tumor necrosis factor receptor superfamily member 14 | Q92956 |  |
| TNFSF13B | Tumor necrosis factor ligand superfamily member 13B | Q9Y275 |  |
| t-PA | Tissue-type plasminogen activator | P00750 |  |
| TR | Transferrin receptor protein 1 | P02786 |  |
| TR-AP | Tartrate-resistant acid phosphatase type 5 | P13686 |  |
| uPA | Urokinase-type plasminogen activator | P00749 |  |
| U-PAR | Urokinase plasminogen activator surface receptor | Q03405 |  |
| vWF | von Willebrand factor | P04275 |  |

**Supplementary Figure 1.** Proposed causal diagram for the association between protein-bound uremic toxins (indoxyl sulfate, p-cresyl sulfate) and cardiovascular protein biomarkers

**
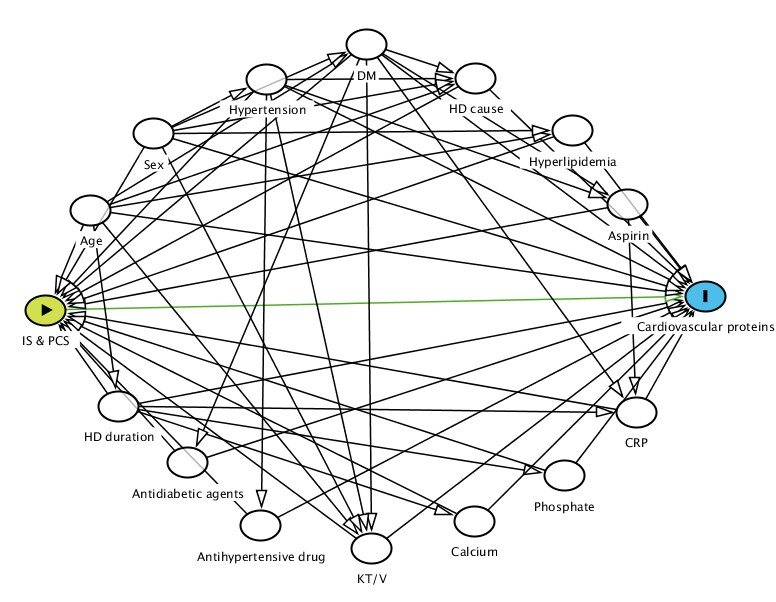
**

**Supplementary Figure 2.** The association between indoxyl sulfate and cardiovascular protein biomarkers in linear regression models with age and sex adjustment

**
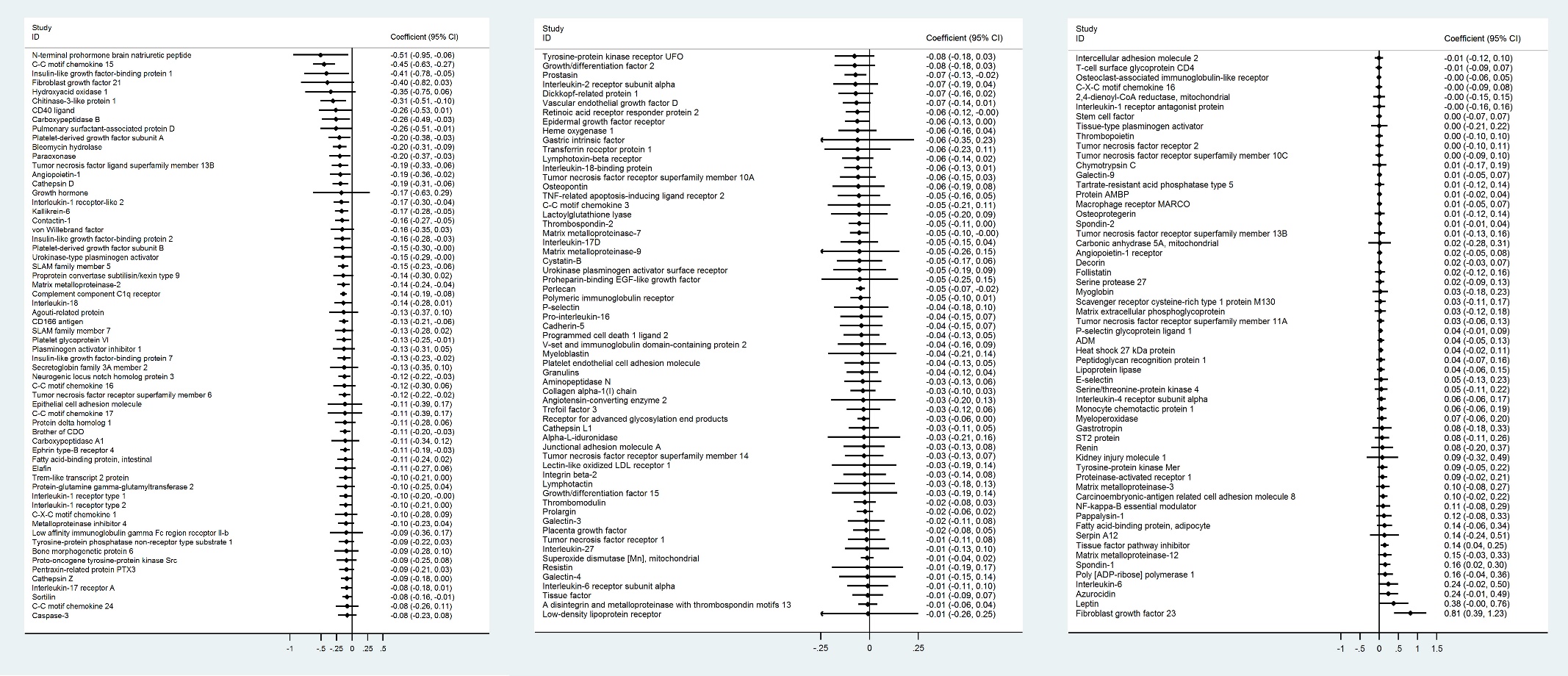
**

**Supplementary Figure 3.** The association between p-cresyl sulfate and cardiovascular protein biomarkers in linear regression models with age and sex adjustment

**
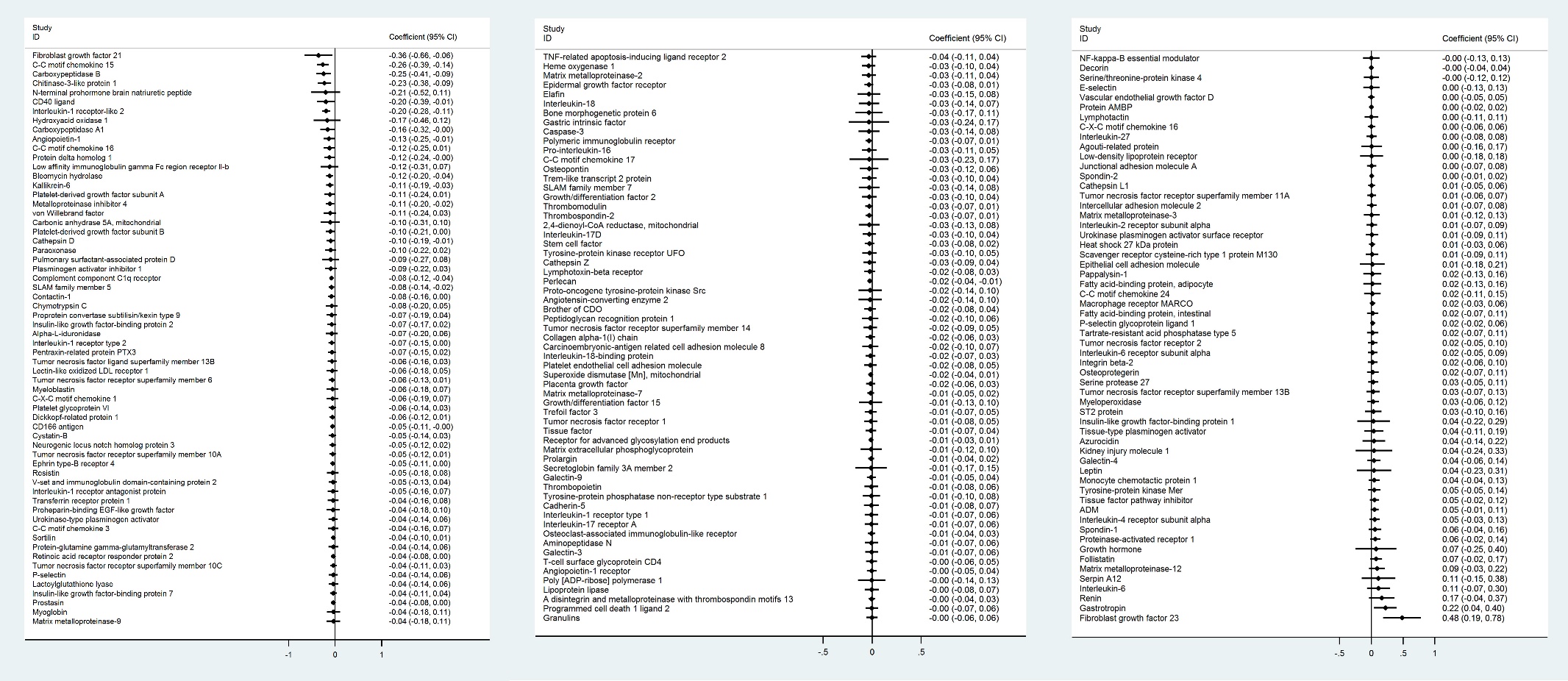
**

**Supplementary Figure 4.** Ranking proteins by p-value with bootstrapped confidence intervals around the ranks related to indoxyl sulfate


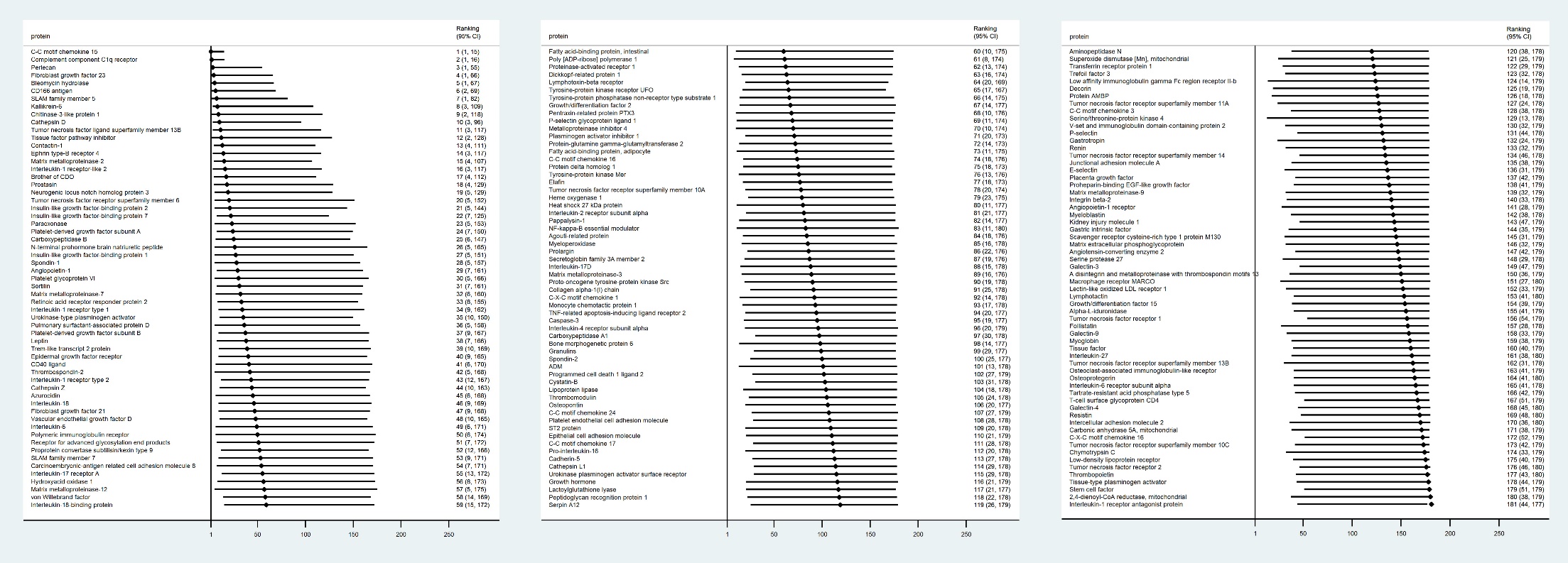


**Supplementary Figure 5.** Ranking proteins by p-value with bootstrapped confidence intervals around the ranks related to p-cresyl sulfate


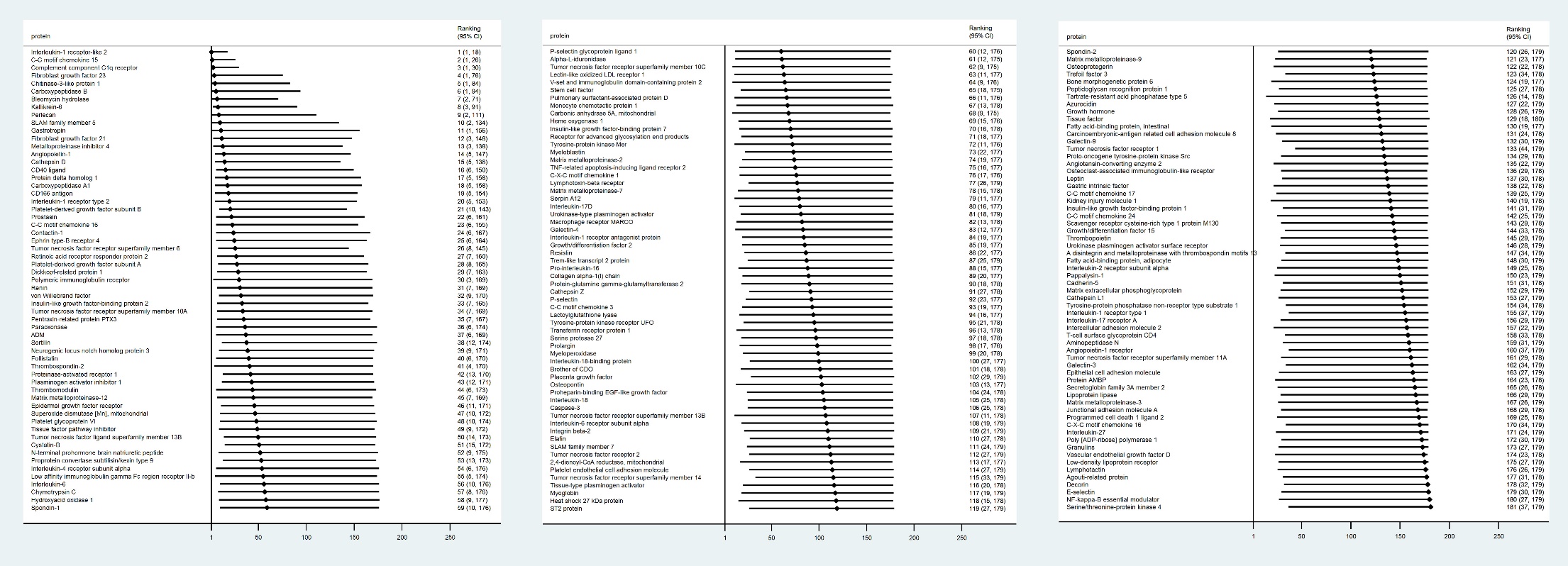


**Supplementary Figure 6.** The cumulative incidence of acute coronary syndrome in patients stratified by median level of cardiovascular proteins (A) C-C motif chemokine 15 (CCL15) (B) Complement component C1q receptor (CD93) (C) Perlecan (D) Bleomycin hydrolase (E) CD166 antigen (F) SLAM family member 5 (CD84) (G) Fibroblast growth factor 23 (FGF23) (H) Interleukin-1 receptor-like 2 (IL1RL2)


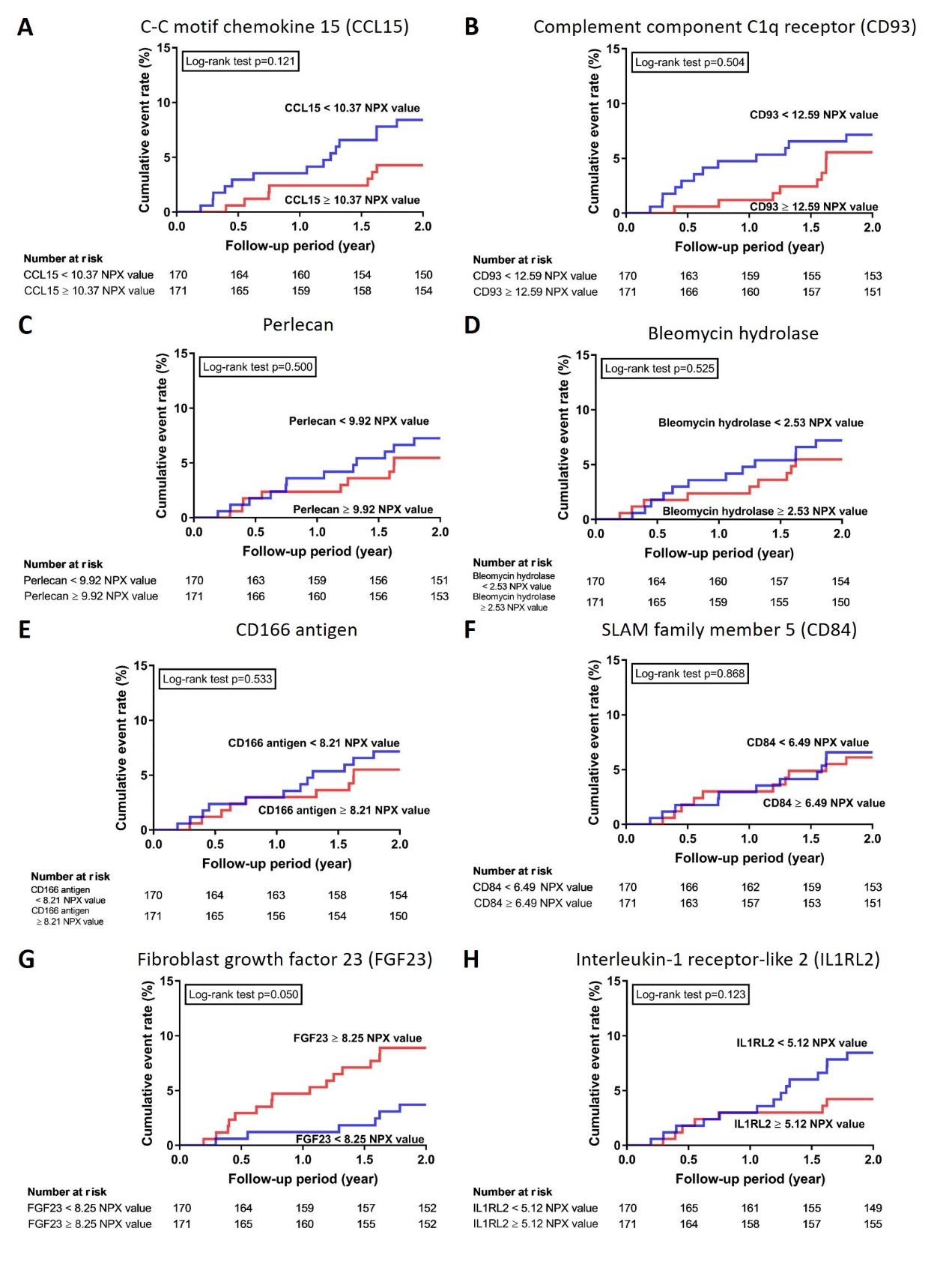

Supplement: Supplementary file 1 — Supplementary Information.[Replace ESM file with the attached 'All Supplementary Tables and Figures (proofreading)_ESM [file 41598_2021_83383_MOESM1_ESM.docx]
